# Superior Pseudocapacitive Storage of a Novel Ni_3_Si_2_/NiOOH/Graphene Nanostructure for an All-Solid-State Supercapacitor

**DOI:** 10.1007/s40820-020-00527-w

**Published:** 2020-10-27

**Authors:** Jing Ning, Maoyang Xia, Dong Wang, Xin Feng, Hong Zhou, Jincheng Zhang, Yue Hao

**Affiliations:** 1 The State Key Discipline Laboratory of Wide Band Gap Semiconductor Technology, Xidian University, Xi’an, 710071 People’s Republic of China; 2 Shaanxi Joint Key Laboratory of Graphene, Xidian University, Xi’an, 710071 People’s Republic of China

**Keywords:** Pseudocapacitive storage, Creeper-like Ni_3_Si_2_, NiOOH, Graphene, All-solid-state supercapacitors

## Abstract

Recent developments in the synthesis of graphene-based structures focus on continuous improvement of porous nanostructures, doping of thin films, and mechanisms for the construction of three-dimensional architectures. Herein, we synthesize creeper-like Ni_3_Si_2_/NiOOH/graphene nanostructures via low-pressure all-solid melting-reconstruction chemical vapor deposition. In a carbon-rich atmosphere, high-energy atoms bombard the Ni and Si surface, and reduce the free energy in the thermodynamic equilibrium of solid Ni–Si particles, considerably catalyzing the growth of Ni–Si nanocrystals. By controlling the carbon source content, a Ni_3_Si_2_ single crystal with high crystallinity and good homogeneity is stably synthesized. Electrochemical measurements indicate that the nanostructures exhibit an ultrahigh specific capacity of 835.3 C g^−1^ (1193.28 F g^−1^) at 1 A g^−1^; when integrated as an all-solid-state supercapacitor, it provides a remarkable energy density as high as 25.9 Wh kg^−1^ at 750 W kg^−1^, which can be attributed to the free-standing Ni_3_Si_2_/graphene skeleton providing a large specific area and NiOOH inhibits insulation on the electrode surface in an alkaline solution, thereby accelerating the electron exchange rate. The growth of the high-performance composite nanostructure is simple and controllable, enabling the large-scale production and application of microenergy storage devices.

**Electronic supplementary material:**

The online version of this article (10.1007/s40820-020-00527-w) contains supplementary material, which is available to authorized users.

## Highlights

- Three types of Ni_3_Si_2_ were successfully fabricated by low-pressure all-solid melting-reconstruction chemical vapor deposition, and the growth pattern changed with the carbon source content.
- In a carbon-rich atmosphere, high-energy atoms bombard the Ni and Si surface, and reduce the free energy in the solid Ni–Si particles’ thermodynamic equilibrium, considerably catalyzing the growth of Ni–Si nanocrystals.
- Ni_3_Si_2_/NiOOH/graphene provides a large specific area and NiOOH inhibits insulation on the electrode surface in an alkaline solution and accelerates the electron exchange rate.

**Electronic supplementary material:**

The online version of this article (10.1007/s40820-020-00527-w) contains supplementary material, which is available to authorized users.

## Graphical abstract

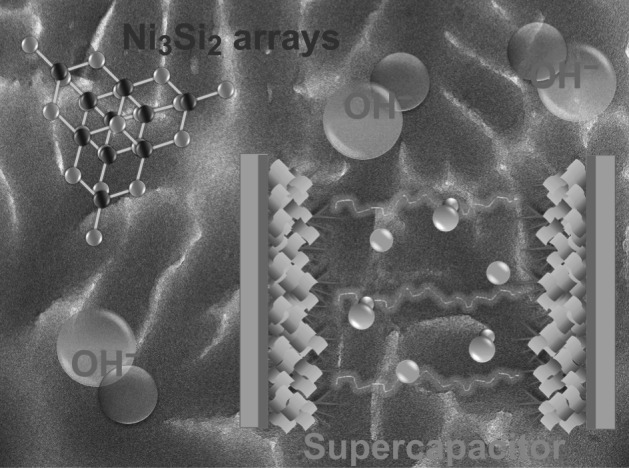

## Introduction

Electrical energy storage systems have become one of the key research objects that are studied to address current energy challenges such as the rapid consumption of fossil fuels, environmental pollution, and global warming [1, 2]. Rechargeable energy storage devices, including traditional capacitors, supercapacitors (SCs), batteries, and fuel cells, are widely used in consumer electronics, transportation, and renewable energy fields [3]. SCs, also called double-layer capacitors, have attracted widespread attention owing to their advantages such as high reliability, fast charge–discharge speed, and high-safety performance [4]. SCs can be classified as: electrical double-layer capacitors and pseudocapacitors [5]. Electrical double-layer capacitor materials, such as three-dimensional (3D) graphene, which have good electrical conductivity and large specific surface area, adsorb electrolyte ions on their surface to store energy [6, 7]. However, the agglomeration and collapse of the 3D structure during operation affect device performance because of the decreased specific surface area [8, 9]. The most effective method to solve this problem is to develop an alternative anode material with a novel structure.

During the nanotechnology revolution of recent decades, one-dimensional nanostructures, such as nanowires, nanotubes, and nanorods, have attracted widespread attention because of their unique physical and chemical properties [10]. The preparation of various nanostructures has significantly progressed using nickel sulfide, which is one of the electrode materials used for lithium batteries [11]. Examples include porous NiS nanoflake arrays [12], Ni_3_S_2_ and Co_9_S_8_ double-size nanoparticles [13], hierarchically structured Ni_3_S_2_/carbon nanotubes [14], Ni_3_S_2_@MoS_2_ core shell nanorods on Ni foam [15], NiS_2_ nanocubes [16], Ni_3_S_2_@Ni composites [17], and Ni_3_S_2_ nanorod arrays on Ni–graphene foams [18]. However, NiS_2_ electrodes have several disadvantages, such as low capacity, poor cycle life, large volume change rate, and solubility in electrolytes, limiting their use in supercapacitors [19]. For example, Pang et al. [16] reported that a NiS_2_ nanocube prepared via the microwave-assisted method has good cycle stability (maintaining 93.4% of the initial specific capacitance after 3000 cycles). However, the specific capacitance of the NiS_2_ nanocube is poor (695 F g^−1^, 1.25 A g^−1^). Wang et al. [20] highlighted that internal stress, which gradually increases as the reaction during the ion insertion/extraction process progresses, cracks, and pulverizes the transition metal sulfide particles. The irreversibility of the electrode material’s microstructural evolution causes the electrode to generate a large irreversible capacity loss in the first cycle. Problems, such as slow ion diffusion and low mobility at the solid–liquid interface, further degrade the performance of the NiS electrode. The most advantageous strategies to solve the above-mentioned problems, including buffering large volume changes of electrodes and improving conductivity, involve the engineering of new nanostructures and the controlled synthesis of new Ni compounds.

Compared with other electrode materials, nickel silicide is advantageous because of its large mechanical stress tolerance, good electrical conductivity, and high electrochemical performance. It is a potential candidate material for supercapacitor electrodes. More importantly, its growth is compatible with and can be easily integrated into silicon process technology [21, 22]. Accordingly, Nickel silicide nanostructures provide a very large surface area because of their unique network structure. In addition, nickel silicide used in binder-free electrodes (the material can be directly grown on a conductive substrate) forms an excellent interface with nickel and exhibits low internal resistance [7]. Jiang et al. [7] reported that Ni_3_Si_2_ nanowires prepared via low-pressure chemical vapor deposition have a high specific capacitance (760 F g^−1^ at 0.5 A g^−1^) and low series resistance (0.93 Ω). By definition, high specific capacitance and low series resistance are key factors to mitigating the tradeoff between the power and energy densities. Therefore, nickel silicide can be used as a new electrode material to solve the limitations of traditional electrical double-layer capacitor materials in supercapacitor applications.

Herein, we synthesized novel creeper-like Ni_3_Si_2_/graphene using a simple and effective low-pressure all-solid melting-reconstruction chemical vapor deposition (LSRCVD) method. The Ni_3_Si_2_/NiOOH/graphene composite nanostructures were synthesized through a hydrothermal method. Based on systematic micromorphological characterization, the formation mechanism of the nanostructures is discussed to develop reliable and controllable nanostructure preparation technology. Under bombardment by high-energy particles in a carbon-rich environment, supersaturation of the Si precursor is decreased by using Ni as the substrate. The silicon vapor is cooled and crystallized on a molten nickel substrate to form Ni–Si nanostructures. By adjusting the carbon content and growth time, we successfully achieved Ni_3_Si_2_ nanocrystal growth in the axial direction and fabricated nanostructures with high surface areas under Ar gas etching. The electrochemical test confirms that the Ni_3_Si_2_/NiOOH/graphene composite exhibits extremely good performance. The specific capacitance of the Ni_3_Si_2_/NiOOH/graphene electrode reaches 835.3 C g^−1^ (1193.28 F g^−1^) at 1 A g^−1^. The fabricated all-solid-state supercapacitor exhibits a maximum energy density of 25.9 Wh kg^−1^ at 750 W kg^−1^. These properties are due to the following reasons: First, the Ni_3_Si_2_/graphene nanoskeleton possesses excellent mechanical strength and supports the entire electrode. Simultaneously, Ni_3_Si_2_ effectively guides the directional deposition of NiOOH with high crystal quality. NiOOH inhibits the insulation of the electrode surface in an alkaline solution and accelerates the electron exchange rate. Second, the interconnected nanostructures have large specific surface areas. Even if parts of the nanoflakes are disconnected from the substrate, the internal electronic circulation will still be protected by physical contact with adjacent nanostructures. Third, in a carbon-rich atmosphere, the surface free energy of solid Ni–Si particles in a thermodynamic equilibrium decreases and excess Ni rapidly diffuses into Ni–Si nanoparticles, which facilitates the formation of Ni–Si nanostructures. Nanoparticles grow in the radial direction to form creeper-like nanostructures. The creeper-like structure increases the specific surface area of the electrode material and the active sites, thereby inducing more redox reactions in the nickel substrate in addition to high conductivity, generating fast charge-transfer kinetics and thus better performance. Fourth, because of the self-supporting 3D porous nanostructure, electrons can traverse the entire electrode surface, creating a convenient electron transfer path for active nanomaterials, thereby improving the supercapacitor performance. The excellent electrochemical properties indicate the potential for integrated SC applications and marketization.

## Experimental Section

### Fabrication of Nanostructures

The creeper-like nanostructures were grown on commercial Ni foam in three steps. First, a hybrid Ni_3_Si_2_/graphene structure was fabricated using a laboratory-made low-pressure chemical vapor deposition (CVD) system. Initially, the surface of the Ni foam was sonicated in a 0.3 mol L^−1^ (NH_4_)_2_S_2_O_8_ solution for 5 min to remove contaminants and oxides. After washing with analytical-grade acetone, ethanol, and deionized water for 5 min each, the Ni foam was dried with nitrogen. Next, the cleaned Ni foam/Si (111) stack was placed into the quartz tube furnace of the CVD system at a base vacuum of ~ 0.1 Pa. Ultrahigh-purity hydrogen was injected into the furnace tube at a flow rate of 10 sccm. Subsequently, the nickel foam was heated from 30 to 1030 °C at a heating rate of 20 °C min^−1^. Once the temperature reached 1030 °C, ultrahigh-purity methane was introduced into the furnace tube (flow rate of 50 sccm) as the carbon source for 2 h. Second, the sample was cooled to 1000 °C and annealed for 1 h in a mixed atmosphere (10 sccm hydrogen, 50 sccm methane and 800 sccm argon). The tube was then slowly cooled to ambient temperature at a cooling rate of ~ 5 °C min^−1^ under 10 sccm of hydrogen flow. Third, the Ni_3_Si_2_/NiOOH/graphene nanostructure was synthesized using a hydrothermal method. The metal foams were selectively etched in a mixture of 1 M FeCl_3_ and 2 M HCl at 60 °C overnight and free-standing Ni_3_Si_2_/graphene was obtained. Subsequently, 100 mg urea was uniformly dispersed in 40 mL deionized water by stirring for 1 h. The mixture was then transferred into a 60-mL Teflon-lined stainless-steel autoclave and the fabricated Ni_3_Si_2_/graphene was added to the mixture. The autoclave was subsequently sealed and maintained at 180 °C for 2 h. Once the reaction was completed, the autoclave was cooled to room temperature. Finally, the sample was clamped with tweezers and washed three times with deionized water and ethanol. The final product was obtained after drying overnight under vacuum at 80 °C. The mass of the graphene, Ni_3_Si_2_, and NiOOH are about 0.18, 0.26, and 0.26 mg, respectively. The detailed analysis process is in the supporting information.

The synthesis process of NiOOH/graphene and Ni_3_Si_2_/graphene is similar to that of Ni_3_Si_2_/NiOOH/graphene, and the detailed process is in the supporting information.

### Nanostructure Characterization

The morphologies of the Ni_3_Si_2_/NiOOH/graphene nanostructures were investigated via scanning electron microscopy (SEM) (Quanta 600FEG, FEI, USA). The crystallinity of the materials was evaluated through high-resolution X-ray diffraction (XRD) (D8 Discovery, Bruker, Germany) in Bragg (reflection) geometry and Cu K_α_1 radiation (wavelength: *λ* = 1.54056 Å). The Raman spectrum was obtained with a Raman spectroscopy system (LabRam HR 800, Horiba JY, Japan) using an Ar^+^ laser (514 nm wavelength) as the excitation source. The elemental analyses of the composites were conducted via X-ray photoelectron spectroscopy (XPS) (ESCALAB, 250Xi, Thermo Fisher Scientific, USA).

### Supercapacitor Fabrication and Evaluation

Cyclic voltammetry (CV), galvanostatic charge/discharge (GCD), and electrochemical impedance spectroscopy (EIS) were performed using the CHI660 electrochemical workstation and a three-electrode test system including the active material as the working electrode, Ag/AgCl as the reference electrode, platinum mesh as the counter-electrode, and 3 M KOH as the electrolyte.

Based on the GCD curves, the specific capacity *Q*_s_ (C g^−1^) and specific capacitance *C*_g_ (F g^−1^) can be computed using Eqs. (1) and (2):

1 $$ Q_{\text{s}} = \frac{I \Delta t}{m  } $$

2 $$ C_{\text{g}} = \frac{I\Delta t}{m \Delta V} $$

where *I* is the discharge current (A), *m* is the total weight (g) and Δ*t* is the discharge time (s).

All SC cell electrochemical measurements were performed using the prepared “sandwich” capacitor. The electrodes, cellulose diaphragms, and electrodes were stacked, and the active materials were in direct contact with the current collector. The electrolyte was gelatinous PVA-KOH.

The specific capacitance (*C*_g_) of the SC cell was calculated using the GCD curves and Eq. (4). The energy and power densities of the SC cell were obtained from Eqs. (3) and (4):

3 $$ E = \frac{1}{2} C_{\text{g}} \Delta V^{2} $$

4 $$ P = \frac{E}{\Delta t} $$

where *E* is the energy density (Wh kg^−1^) and *P* is the power density (W kg^−1^).

## Results and Discussion

### Characterizations

**Fig. 1:**
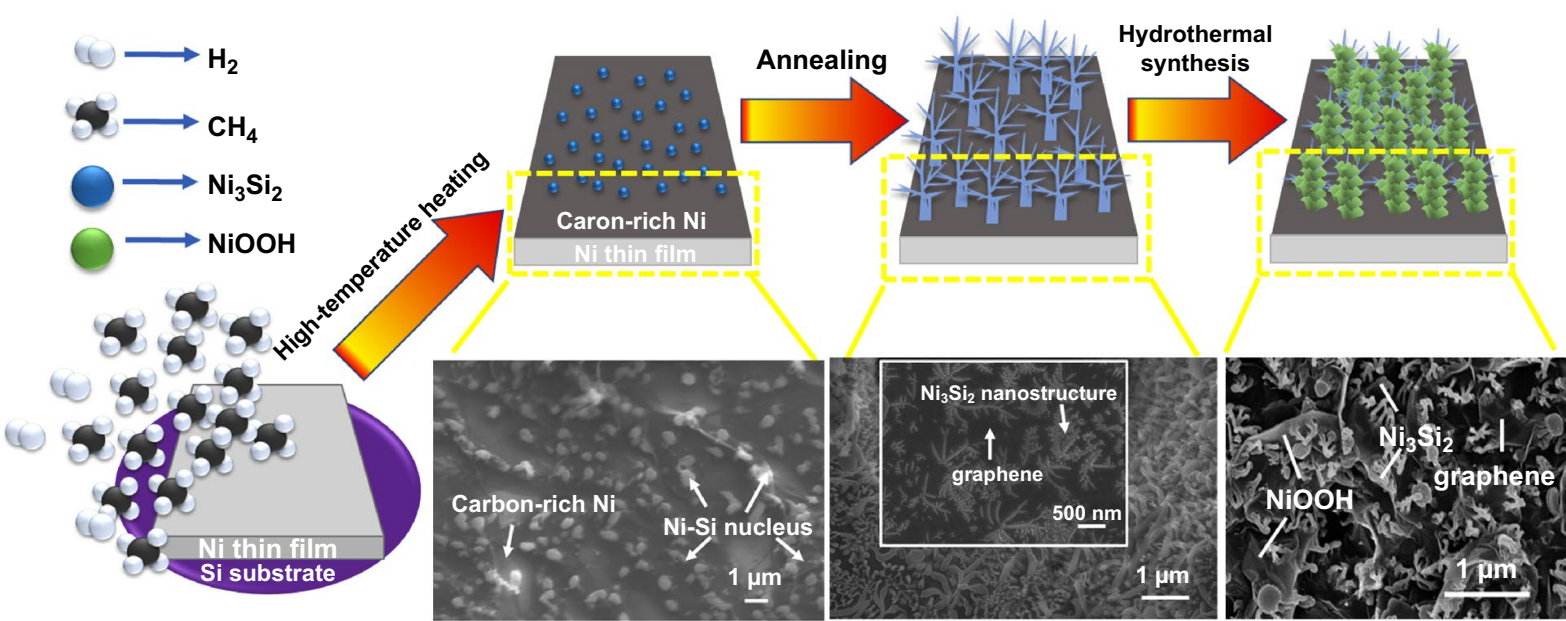
Schematic diagram of the formation and evolution of creeper-like nanostructures

To ensure high purity and controllability, novel creeper-like Ni_3_Si_2_/NiOOH/graphene nanostructures were synthesized on 3D graphene/Ni foam using a three-step method. Figure 1 schematically illustrates the formation mechanism of the creeper-like Ni_3_Si_2_/NiOOH/graphene nanostructures.

In the first step, CH_4_ was decomposed through catalysis with the Ni foam and the carbon atoms dissolved in the 3D Ni foam. Simultaneously, the Si vapor mixed with Ni vapor in a carbon-rich environment, the formation of Ni–Si nanocores form from supersaturation of Ni/Si vapor, the nanocore melting, cooled and then condensed on the surface of the Ni foam. Several Ni–Si nanocores formed during condensation. In the second step, carbon atoms precipitated from the Ni foam and were transferred into graphene along the 3D Ni foam surface during annealing. During melting and condensation, the Ni–Si nuclei randomly merged and flowed on the graphene surface. Then, these nuclei grew radially and axially, forming a creeper-like nanoskeleton. In the third step, several Ni_3_Si_2_ units on the surface reacted with urea during the hydrothermal process to form NiOOH nanosheets, which dissolved in the solution. Ni_3_Si_2_ acts as the nanoframework to guide the deposition of NiOOH nanosheets, and a creeper-like nanostructure forms through the complex directional adhesion process.

**Fig. 2:**
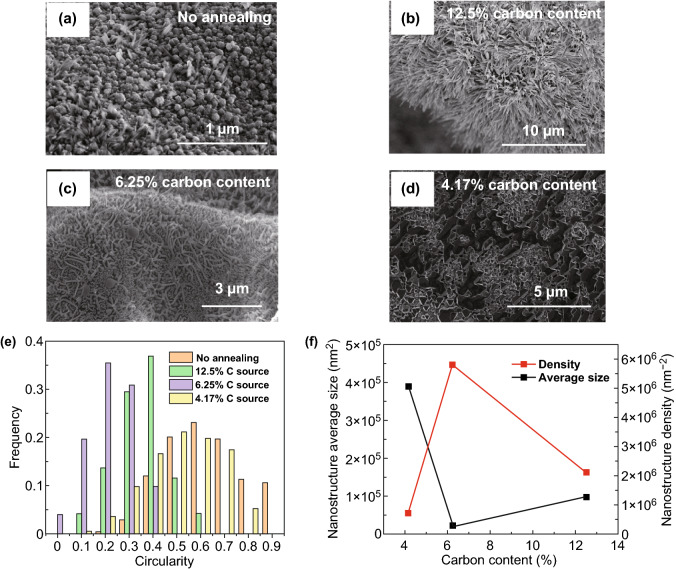
**a** Scanning electron microscopy (SEM) image of nanocore Ni_3_Si_2_ nanostructure. **b** SEM image of nanowire Ni_3_Si_2_ nanostructure. **c** SEM image of creeper-like Ni_3_Si_2_ nanostructure. **d** SEM image of amorphous Ni_3_Si_2_ nanostructure. **e** Circularity frequency distribution. **f** Time-dependent variation in the average size and density of creeper-like Ni_3_Si_2_/NiOOH/graphene nanostructures on 3D graphene/Ni foam. The images were obtained at different carbon contents during annealing

The carbon content is one of the main factors affecting the synthesis of Ni_3_Si_2_ nanostructures using LSRCVD. The way to control the carbon content is to change the Ar flow rate. In the experiment, 12.5% carbon content atmosphere includes 10 sccm H_2_, 50 sccm CH_4_, 340 sccm Ar, 6.25% carbon content atmosphere includes 10 sccm H_2_, 50 sccm CH_4_, 740 sccm Ar, and 4.17% carbon content atmosphere includes 10 sccm H_2_, 50 sccm CH_4_, 1140 sccm Ar. During annealing, Ni–Si nanocrystals start to grow; the gas flow rate strongly affects the growth of the nanostructures, as shown in Fig. 2. After high-temperature heating, a large amount of irregular Ni–Si dust (average size of ~ 6000 nm^2^) condensed and crystallized as the crystal nucleus on the surface of the Ni foam (Fig. 2a). In the carbon-rich environment (carbon content > 10%), the Ni_3_Si_2_ nanostructure followed a radial growth model (Fig. 2b). In the radial direction, a small amount of Si vapor condensed owing to the large amount of CH_4_, the supersaturation of Ni vapor was high, and the surface free energy of the Ni–Si core in thermodynamic equilibrium was reduced. Thermally activated Ni atoms continuously and rapidly diffused to the Ni–Si nucleation center and reached a certain degree of supersaturation, resulting in the growth of columnar roots in the Ni–Si nanocrystals. With decreasing carbon content (~ 10%), the crystallization mechanism changed to predominant axial growth (Fig. 2c). The variation of the surface free energy becomes small because of the decrease in Si and Ni vapor saturation, rendering the radial diffusion of Ni atoms difficult. The Ni–Si dust melted into droplets and subsequently coalesced. Ni–Si droplets with long tails formed in different directions on the surface of the Ni foam. At a very low carbon content (< 5%), the radial growth of Ni–Si nanostructures was completely inhibited. The evaporated Si and Ni were quickly removed by Ar. The thin film on the surface relied on the combination of Ni–Si dust and a porous structure formed under the etching of Ar (Fig. 2d). Figure 2e, f shows the effects of the carbon content on the morphology, size, and density of the Ni_3_Si_2_ crystals. With reduction in the carbon content, the crystal morphology changed from nanowire- to creeper-shaped, and the crystals finally became amorphous (SEM, TEM and EDS images of amorphous Ni_3_Si_2_ are shown in Fig. S2). Because decreased number of carbon atoms increased the required surface free energy, Ni–Si nucleation became difficult. Figure 2f shows that the density and size reached extreme values at a carbon content of 6.25%. The largest specific area is observed under these conditions. These results demonstrate the validity of the above-mentioned nucleation theory. Therefore, carbon regulation can be used to control the morphology, size, and density of crystals.

**Fig. 3:**
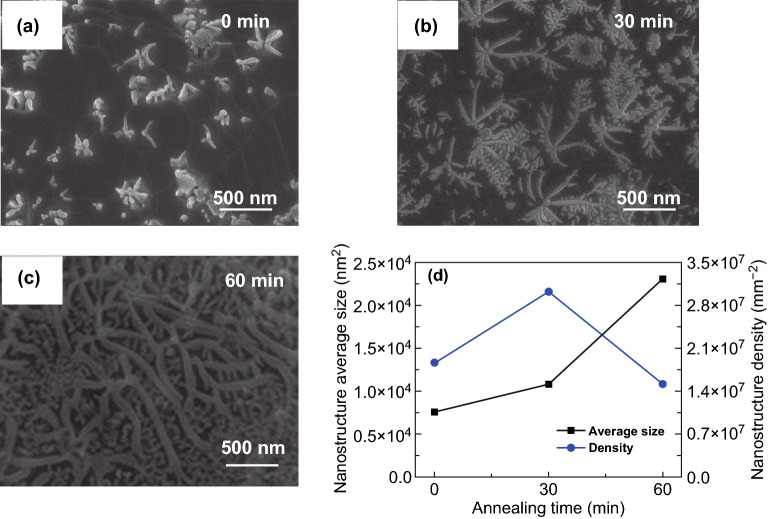
SEM images of Ni-Si nanostructures for growth periods **a** of 0 min with 6.25% carbon content, **b** of 30 min with 6.25% carbon content, **c** of 60 min with 6.25% carbon content. **d** Time-dependent variation in the average size and density of nanostructures

The annealing time is another key factor affecting the fabrication of Ni_3_Si_2_ nanostructures using LSRCVD. Figure 3 shows SEM images of the Ni_3_Si_2_ nanostructures for different growth times with 6.25% carbon content. Initially, mostly incomplete nanostructures were formed. In addition, the densities of the nanostructures were low, as shown in Fig. 3a. With the increase in the annealing time from 0 to 30 min, the densities and average sizes of the creeper-like nanostructures gradually increased. After 30 min, the densities of the creeper-like nanostructures decreased; however, the average sizes continued to increase (Fig. 3b, c). At the beginning of annealing, the Ni–Si content was low. The Ni and Si vapors continuously condensed on the Ni surface, producing enough nuclei and growth. With increasing nuclear density, the space for nanostructure nucleation decreased, and several nanoflakes merged to form large-scale nanostructures. However, the growth mechanism controlled by the diffusion of the solid phase requires a specific particle size range, which limits the nanostructure size. Therefore, the average size and nuclear density appear to be competitive, which provides the possibility of controlled Ni_3_Si_2_ nanostructure growth.

**Fig. 4:**
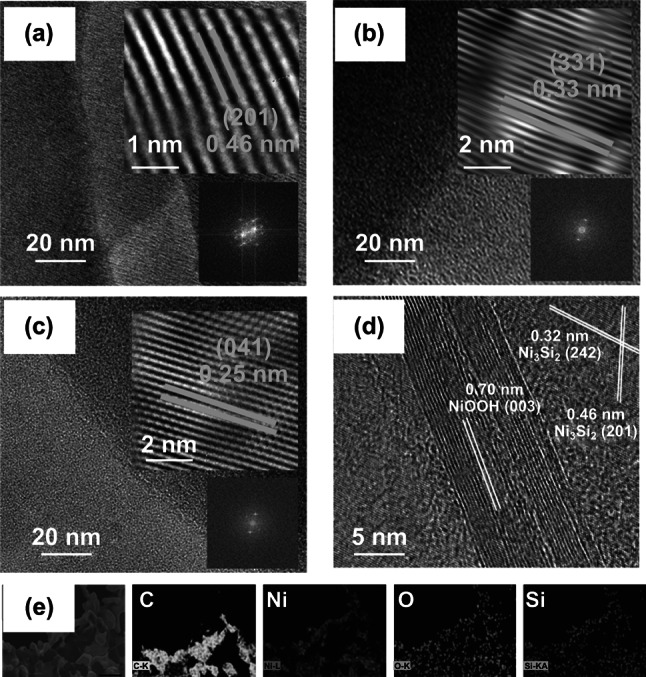
**a**–**d** TEM images of Ni_3_Si_2_/NiOOH/graphene creeper-like nanostructures. **e** EDS scans of Ni_3_Si_2_/NiOOH/graphene creeper-like nanostructures

Figure 4 shows the TEM (inset: schematic structures of Ni_3_Si_2_/NiOOH/graphene) and EDS images of the Ni_3_Si_2_/NiOOH/graphene creeper-like nanostructures. The lattice fringe distances of 0.25, 0.32, 0.33, and 0.46 nm in Fig. 4b–d can be attributed to the (041), (242), (331), and (201) planes of the Ni_3_Si_2_ structure, respectively [22]. And the lattice fringe distance of 0.70 nm is attributed to the (003) planes of the NiOOH [23, 24]. Figure 4e shows that the creeper-like nanostructures contain uniformly distributed C, Ni, O, and Si.

**Fig. 5:**
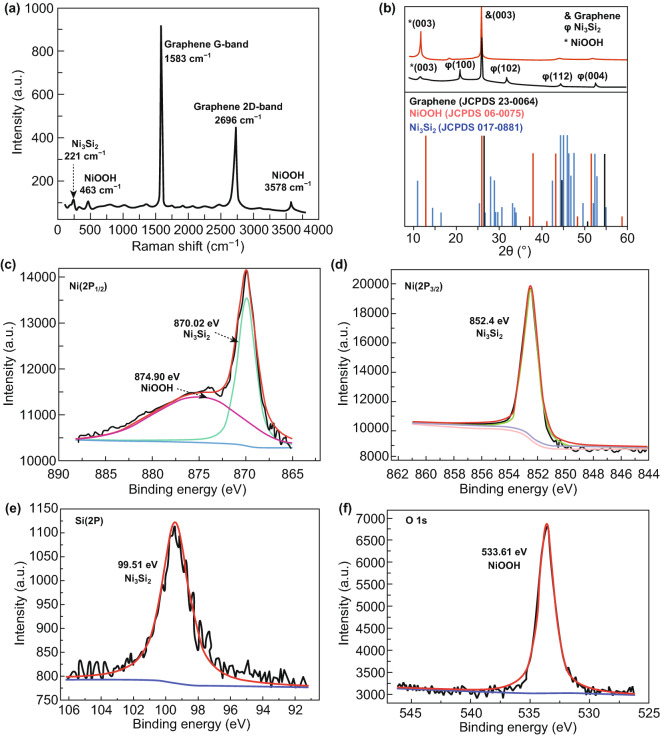
**a** Raman spectra of Ni_3_Si_2_/NiOOH/graphene. **b** XRD patterns of NiOOH/graphene and Ni_3_Si_2_/NiOOH/graphene. **c–f** XPS survey spectra of Ni_3_Si_2_/NiOOH/graphene electrodes (Ni 2*p*, Si 2*p*, and O 1*s*)

Raman spectroscopy was conducted to investigate the crystalline structure of the Ni_3_Si_2_/NiOOH/graphene (Fig. 5a). The Raman spectrum of graphene shows two main vibration bands at 1583 and 2696 cm^−1^, corresponding to the G and 2D bands of graphene, respectively. Compared with that of the graphene peaks [25], the peaks in Raman spectra of Ni_3_Si_2_ and NiOOH indicate a clearly distinct set of low-intensity vibration modes. The band at 221 cm^−1^ belongs to Ni_3_Si_2_ and represents the excitation band of a single Ni_3_Si_2_ phase [26]. The bands at 463 and 3578 cm^−1^ are attributed to NiOOH [27].

Typical XRD patterns of the Ni_3_Si_2_/NiOOH/graphene nanostructure and NiOOH/graphene are shown in Fig. 5b. The high-intensity peak at 26.5° is related to the (002) crystal plane of graphene (JCPDS 23-0064). The diffraction peak at 12.8° corresponds to the (003) plane of NiOOH (JCPDS 06-0075). The characteristic peaks at 23.3°, 33.2°, 44.9°, and 52.8° are attributed to the (310), (330), (242), and (004) crystal planes of Ni_3_Si_2_ (JCPDS No. 017-0881), respectively. Compared with that of NiOOH/graphene, the intensity of the graphene and NiOOH peaks is much stronger than that of Ni_3_Si_2_/NiOOH/graphene, which could be caused by the presence of unrepaired defects such as lattice mismatch.

The chemical compositions of the Ni_3_Si_2_/NiOOH/graphene materials were characterized via XPS. The survey scan spectra (Fig. 5c–f) exhibit Ni 2*p*_1/2_, Ni 2*p*_3/2_, Si 2*p*, and O 1*s* peaks. The Ni 2p_1/2_ spectrum in Fig. 5c shows two major peaks with binding energies of 870.02 and 874.9 eV, which correspond to Ni_3_Si_2_ and NiOOH, respectively. The Ni 2*p*_3/2_ spectrum in Fig. 5d shows a peak at 852.4 eV, which is due to Ni_3_Si_2_. The Si 2*p* peak at 99.51 eV corresponds to Ni_3_Si_2_ (Fig. 5e). The strong peak at 533.6 eV in the O 1*s* spectrum is due to NiOOH (Fig. 5f).

### Electrochemical Characterization

To determine the electrochemical performance of the positive electrode, cyclic voltammetry (CV), galvanostatic charge/discharge (GCD), and electrochemical impedance spectroscopy (EIS) of the Ni_3_Si_2_/NiOOH/graphene were performed in a 3 M KOH solution using a three-electrode system. We combined nanowire-shaped, creeper-shaped and amorphous Ni_3_Si_2_/graphene with NiOOH, respectively, denoted as Ni_3_Si_2_/NiOOH/graphene-1, Ni_3_Si_2_/NiOOH/graphene-2 and Ni_3_Si_2_/NiOOH/graphene-3. From Fig. S5a, the Ni_3_Si_2_/NiOOH/graphene-2 electrodes show considerably largest enclosed areas, which indicates that electrochemical capacitance is improved owing to pseudocapacitive contributions. Furthermore, for the GCD measurements (Fig. S5b), Ni_3_Si_2_/NiOOH/graphene-1, -2 and -3 electrodes clearly differ owing to the significant contributions from Faradaic capacitance. The considerably extended discharge time of Ni_3_Si_2_/NiOOH/graphene-2 again proves their enhanced capacitance. The Nyquist plots obtained by EIS are composed of an arc in the high-frequency region and a straight line in the low-frequency region, which can be simulated by an equivalent circuit (Fig. S5c). Obviously, Ni_3_Si_2_/NiOOH/graphene-2 has minimal series (0.28 Ω) and contact resistances (0.8 Ω), which indicates better capacitance performance. It attributed to creeper-like Ni_3_Si_2_/graphene nanostructure has smaller crystal size and higher density. Electrolyte ions fully contact with the electrode and increase the effective specific surface area, and the creeper-like Ni_3_Si_2_/NiOOH/graphene electrode provides more active sites to store energy.

**Fig. 6:**
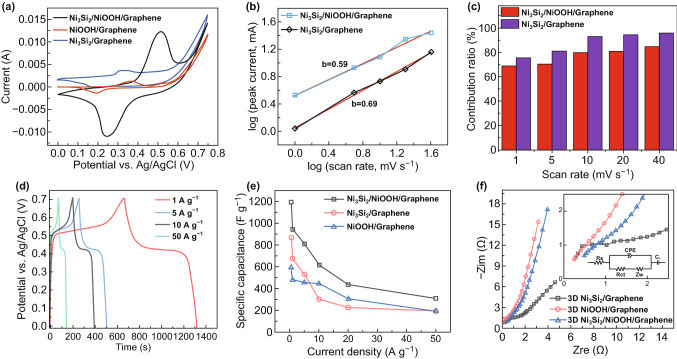
**a** Cyclic voltammetry (CV) curves at 10 mV s^−1^. **b** Log (scan rate) versus log (peak current) graph for the anodic peaks. **c** Pseudocapacitive contribution (%) at different scan rates. **d** Galvanostatic charge/discharge (GCD) curves at various current densities. **e** Specific capacitance depending on the current density. **f** Nyquist plots of Ni_3_Si_2_/NiOOH/graphene, Ni_3_Si_2_/graphene, and NiOOH/graphene

Figure 6a shows the comparison between different CV curves obtained at a scan rate of 10 mV s^−1^. The area of the CV curve of the electrode with Ni_3_Si_2_/NiOOH/graphene is larger than that of NiOOH/graphene and Ni_3_Si_2_/graphene, which indicates high capacitance. Notably, the three CV curves show different redox peaks at the same scanning rate because the Gibbs free energy of the surface redox reaction of Ni_2*p*_/Ni_3*p*_ differs. The relative redox process can be described using Eqs. (5) and (6):

5 $$ {\text{NiOOH }} + {\text{H}}_{2} {\text{O }} + {\text{e}}^{ - } = {\text{Ni}}\left( {\text{OH}} \right)_{2} + {\text{OH}}^{ - } $$

6 $$ {\text{Ni}}_{3} {\text{Si}}_{2} + 3{\text{OH}}^{ - } = {\text{Ni}}_{3} {\text{Si}}_{2} ({\text{OH}})_{3} + 3{\text{e}}^{ - } $$

With increasing scan rate, the current response increases concomitant with the shift of the redox peaks; thus, the Faradic reaction may be controlled by ion diffusion. The storage mechanisms of the electrodes were studied by analyzing the relationship between the peak current (*i*) and scan rate (*ʋ*) using Eqs. (7) and (8):

7 $$ i = a\upsilon^{b} $$

8 $$ \log \left( i \right) = \log \left( a \right) + b\log \left( \upsilon \right) $$

The *b* value generated by the slope can be used to determine the control mechanism of the electrode. Typically, *b* values close to 1.0 and 0.5 indicate pseudocapacitive behavior and a battery (diffusion-controlled) process, respectively. As shown in Fig. 6b, the *b* values for the anode peaks in Ni_3_Si_2_/graphene and Ni_3_Si_2_/NiOOH/graphene are 0.69 and 0.59, respectively, indicating that the specific capacitances of the electrodes are based on both diffusion-controlled and pseudocapacitive behaviors. Adding NiOOH increases the size of the nanostructure and proportion of the current generated by the volume response. Therefore, the Ni_3_Si_2_/NiOOH/graphene electrode mainly exhibits diffusion control. The pseudocapacitive contribution can be investigated using the CV curves and Eqs. (9) and (10):

9 $$ i\left( V \right) = k_{1} v + k_{2} v^{0.5} $$

10 $$ \frac{i\left( V \right)}{{v^{0.5} }} = k_{1} v^{0.5} + k_{2} $$

where *k*_1_ and *k*_2_ are changeable parameters originating from the slope and *y*-axis intercept of the curves between *i ν*^−0.5^ and *ν*^0.5^. The parameters *k*_1_ *ν* and *k*_2_ *ν*^0.5^ represent the fractions of the capacitive and diffusion-controlled behaviors, respectively. The pseudocapacitive contribution fractions are shown in Fig. 6c. Based on the above-mentioned calculation, the pseudocapacitive contributions obtained for Ni_3_Si_2_/graphene are 75.6%, 81.1%, 93.0%, 94.4%, and 95.5% at scan rates of 1, 5, 10, 20, and 40 mV s^−1^, respectively. Similarly, the ratios of the pseudocapacitive contribution of Ni_3_Si_2_/NiOOH/graphene are 69.1%, 70.3%, 79.8%, 80.8%, and 84.4% at scan rates of 1, 5, 10, 20, and 40 mV s^−1^, respectively. At high scan rates, the pseudocapacitive storage behavior is outstanding because fast ion intercalation/deintercalation produces good reversibility and rate capability.

In addition, the GCD curves of Ni_3_Si_2_/NiOOH/graphene are shown in Fig. 6d. The GCD curves show pairs of voltage platforms during the long charge–discharge process, which indicates a superior Faradic efficiency and excellent reversibility. From the plot of Coulombic efficiency (Fig. S3), the Coulomb efficiency is 95.52% at a current density of 1 A g^−1^. Even at a high current density of 50 A g^−1^, the Coulomb efficiency can reach 86.93%. The specific capacity of the device at a current density of 1 A g^−1^ calculated from the discharge branch of the device is 835.3 C g^−1^ (1193.28 F g^−1^). The specific capacitance values of the electrodes were calculated based on their corresponding GCD curves, as shown in Fig. 6e. At the same current density, the performance of the Ni_3_Si_2_/NiOOH/graphene electrode is much better than that of the Ni_3_Si_2_/graphene, and NiOOH/graphene electrodes because adding NiOOH provides more active sites and accelerates the ion exchange of the electrode surface in alkaline solution. The electrons react with NiOOH on the surface to generate Ni(OH)_2_ and OH^−^. Then, OH^−^ reacts with Ni_3_Si_2_ to generate electrons and Ni_3_Si_2_(OH)_3_. It is a positive feedback process until the electrode material is transformed into Ni(OH)_2_ and Ni_3_Si_2_(OH)_3_. Additionally, the unique spatial structure of Ni_3_Si_2_ eases the agglomeration and deformation of NiOOH.

The Nyquist plots of Ni_3_Si_2_/graphene, NiOOH/graphene, and Ni_3_Si_2_/NiOOH/graphene are shown in Fig. 6f. The charge-transfer resistance of the NiOOH/Graphene electrode, Ni_3_Si_2_/Graphene electrode, and Ni_3_Si_2_/NiOOH/Graphene electrode are 0.4, 0.97, and 0.36 Ω, respectively. The equivalent series resistance is 0.23, 0.28, and 0.47 Ω for the NiOOH/Graphene electrode, Ni_3_Si_2_/Graphene electrode, and Ni_3_Si_2_/NiOOH/Graphene electrode, respectively. Ni_3_Si_2_/NiOOH/graphene has the largest equivalent series resistance, which is due to the lattice mismatch at the interface of Ni_3_Si_2_ and NiOOH. In an alkaline environment, a protective layer of SiO_2_·2H_2_O and/or Ni_2_SiO_4_ can form on the surface of Ni_3_Si_2_. Because Ni_2_SiO_4_ and SiO_2_·2H_2_O are insulators, the charge transfer and diffusion processes on the electrode surface are suppressed [28]. Therefore, Ni_3_Si_2_/graphene has the largest Warburg and charge-transfer resistances. NiOOH has excellent conductivity and prevents insulator production on the electrode surface, which considerably reduces difficulties in transporting charges. In the low-frequency region, the slope of the straight line indicates the difficulty of ion diffusion. The closer the angle between the line and the *x*-axis is to 90°, the higher the transmission efficiency of surface ions. The angle of the NiOOH/Graphene electrode, Ni_3_Si_2_/Graphene electrode, and Ni_3_Si_2_/NiOOH/Graphene electrode are 83.48°, 60.53°, and 79.70°. Overall, Ni_3_Si_2_/NiOOH/graphene has faster kinetics, higher conductivity, and a lower ion diffusion resistance during the charge–discharge process.

### Ni_3_Si_2_/NiOOH/graphene Symmetric Supercapacitors

**Fig. 7:**
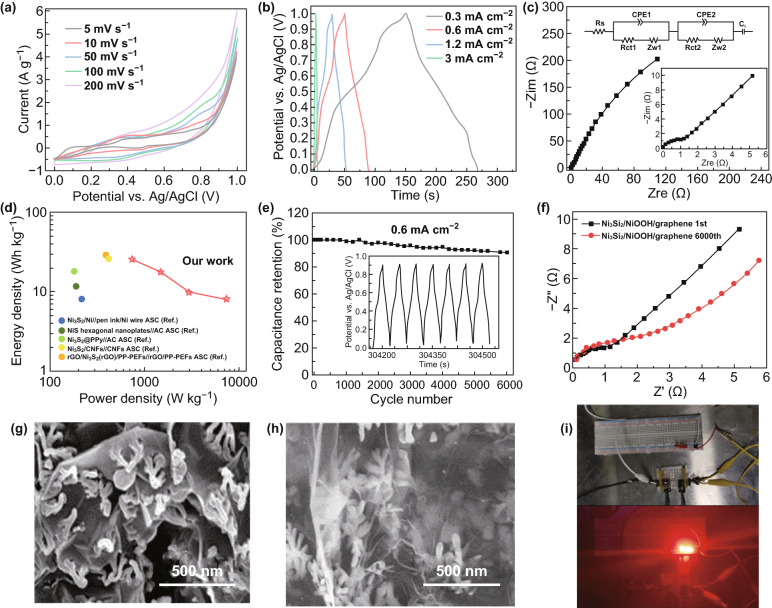
**a** CV curves of the all-solid-state Ni_3_Si_2_/NiOOH/graphene supercapacitor (SC). **b** GCD curves of the all-solid-state Ni_3_Si_2_/NiOOH/graphene SC. **c** Nyquist plots of the all-solid-state Ni_3_Si_2_/NiOOH/graphene SC. **d** Ragone plot of the all-solid-state Ni_3_Si_2_/NiOOH/graphene SC. **e** Cyclic lifetime of the all-solid-state Ni_3_Si_2_/NiOOH/graphene SC. **f** Nyquist plot of the Ni_3_Si_2_/NiOOH/graphene electrode before and after 6000 cycling. **g** SEM images of the Ni_3_Si_2_/NiOOH/graphene electrode before cycling. **h** SEM images of the Ni_3_Si_2_/NiOOH/graphene electrode after 6000 cycling at 0.6 mA cm^−2^. **i** Device connection diagram and the device to light up a LED light

Ni_3_Si_2_/NiOOH/graphene was chosen to assemble symmetrical all-solid-state SCs and explore their energy storage performances. Figure 7a displays the CV curves of an all-solid-state SC at different scan rates and a voltage window ranging from 0 to 1.0 V. The volt–ampere cycle curve is rectangular and shows a wide peak. Electrolyte ions undergo a rapid and highly reversible process of insertion and removal on the electrode surface, which represents the mechanism of ion intercalation in a Faraday quasi-capacitor. Figure 7b outlines the charge–discharge storage capacity of the devices based on the GCD at different current densities. The shape of the GCD curve is symmetrical and a charge–discharge platform appears during the charge–discharge process, indicating that the device exhibits pseudocapacitive behavior. The specific capacities are 186.5 (186.5 F g^−1^), 126.3, 71.4, and 58.5 C g^−1^ at 0.3, 0.6, 1.2, and 3 mA cm^−2^, respectively. Figure 7c shows the Nyquist plots of the all-solid-state SCs. The equivalent series resistance of the capacitors is ~ 0.1 Ω and the charge-transfer resistance is ~ 0.5 Ω. The large slope in the low-frequency region indicates that the Warburg impedance is minimal. The energy and power densities of the all-solid-state SC were calculated according to the discharged branches of the GCD curves (Fig. 7d). The maximal energy density is 25.9 Wh kg^−1^ at 750 W kg^−1^, and the maximal power density is 7500 W kg^−1^ at 8.13 Wh kg^−1^. Compared with previous reports [29–33], the Ni_3_Si_2_/NiOOH/graphene all-solid-state SC exhibits higher energy and power densities. Therefore, the Ni_3_Si_2_/NiOOH/graphene all-solid-state SC can store more energy per unit weight and output a larger current density. Figure 7e shows the cyclic stability of all-solid-state capacitors. At a current density of 0.6 mA cm^−2^, 90.7% of the capacitance is maintained after 6000 cycles. From Fig. 7g, h, after 6000 charge and discharge cycles, the Ni_3_Si_2_ structure has not changed significantly, it demonstrates the durability of the Ni_3_Si_2_/NiOOH/graphene nanostructure. But a thin film is formed on the surface of the electrode to wrap the entire electrode. Figure 7f shows that due to the existence of the thin film, the contact resistance and Warburg resistance of the electrode increase. It indicated that the thin film reduces the active sites on the electrode surface and reduces the electrolyte ion exchange efficiency. In Fig. 7i, we connected two Ni_3_Si_2_/NiOOH/graphene supercapacitors in series and charge the supercapacitor system to 2 V. When the voltage across the system reaches 2 V, disconnect the charging system and drive the LED light. In addition, Ni_3_Si_2_/NiOOH/graphene demonstrates superior specific capacitance by comparison with other materials (Table 1). Generally, the Ni_3_Si_2_/NiOOH/graphene all-solid-state SC has impressive storage and output capabilities and can be applied in applications such as integrated circuits.

**Table 1:** Electrochemical properties of different nickel compounds and silicides and their composites as electrode materials

Electrode materials	Electrolyte	Specific capacitance (F g^−1^)	Cycle	Energy density (Wh kg^−1^)	Refs.
NiOOH-decorated α-FeOOH	1 M KOH	554.17 (at 1.1 A g^−1^)	82.61% (4000 cycles)	–	[34]
Ni(OH)_2_/NG	6 M KOH	1350 (at 2 A g^−1^)	83.08% (1000 cycles)	–	[35]
Ni/GO	6 M KOH	461 (at 5 mV s^−1^)	98.05% (1000 cycles)	–	[36]
H-NiOOH/GS	2 M KOH	1162 (at 1 A g^−1^)	85.3% (8000 cycles)	66.8 (800 W kg^−1^)	[37]
CoNiSi/C	3 M KOH	226 (at 0.5 A g^−1^)	99% (10,000 cycles)	20 (94.5 W kg^−1^)	[38]
NiSi hollow sphere	3 M KOH	66.7 (at 0.5 A g^−1^)	44% (5000 cycles)	3.78	[39]
NiSi–Ni(OH)_2_	3 M KOH	476.4 (at 2 A g^−1^)	103% (10,000 cycles)	21.6 (413.7 W kg^−1^)	[40]
Nickel silicide nanowires	2 M KOH	187.92 (at 2 A g^−1^)	79% (3000 cycles)	13.37 (200 W kg^−1^)	[41]
Ni_3_Si_2_ nanowires	2 M KOH	760 (at 0.5 A g^−1^)	60% (1000 cycles)	17.5 (301 W kg^−1^)	[7]
Ni_3_Si_2_/NiOOH/graphene	3 M KOH	1193.28 (at 1 A g^−1^)	90.7% (6000 cycles)	25.9 (750 W kg^−1^)	Our work

## Conclusions

In summary, we successfully fabricated novel creeper-like Ni_3_Si_2_/graphene nanostructures using LSRCVD based on which Ni_3_Si_2_/NiOOH/graphene nanostructures were hydrothermally synthesized. Based on the SEM images, the Ni_3_Si_2_/graphene nanostructures formed in a carbon-rich atmosphere by melting the surface of Ni foam and condensing the resulting vapor, by growth of 3D graphene by Ni, and by nucleation of Ni–Si. By adjusting the growth temperature and duration, the nucleation and evolution of these creeper-like nanostructures can be precisely controlled. The electrochemical measurement results show that creeper-like Ni_3_Si_2_/NiOOH/graphene nanostructures exhibit excellent performances and are suitable for use as energy storage materials. The charge storage mechanism of Ni_3_Si_2_/NiOOH/graphene used as SC electrode is primarily determined by two types of Ni_2p_/Ni_3p_ pseudocapacitive reactions. Creeper-like nanostructured electrodes provide a high specific capacitance (1193.28 F g^−1^ at 1 A g^−1^). The all-solid-state SCs based on Ni_3_Si_2_/NiOOH/graphene nanostructures exhibit outstanding supercapacitor performance, exhibiting an extremely high-energy density (25.9 Wh kg^−1^ at 750 W kg^−1^). Even after 6000 cycles at 0.6 mA cm^−2^, only 10% of the capacitance is lost. This impressive electrochemical behavior indicates that creeper-like Ni_3_Si_2_/NiOOH/graphene nanostructures have great potential in the energy storage field.

## Electronic supplementary material

Below is the link to the electronic supplementary material.

Supplementary material 1 (PDF 629 kb)
