# Supplementary material for: Superior Pseudocapacitive Storage of a Novel Ni3Si2/NiOOH/Graphene Nanostructure for an All-Solid-State Supercapacitor
Source: Nanomicro Lett. 2020 Oct 27;13:2. doi: 10.1007/s40820-020-00527-w (PMC8187555; doi:10.1007/s40820-020-00527-w)
Supplement: Supplementary file 1 — Supplementary material 1 (PDF 629 kb) [file 40820_2020_527_MOESM1_ESM.pdf]

Supporting Information for

Superior Pseudocapacitive Storage of a Novel Ni_3Si_2/NiOOH/Graphene Nanostructure for an All-Solid-State Supercapacitor

Jing Ning[1, 2, *], Maoyang Xia[1, 2], Dong Wang[1, 2, *], Xin Feng[1, 2], Hong Zhou[1, 2], Jincheng Zhang[1, 2], Yue Hao[1, 2]

[1]The State Key Discipline Laboratory of Wide Band Gap Semiconductor Technology, Xidian University, Xi'an 710071, People's Republic of China

[2]Shaanxi Joint Key Laboratory of Graphene, Xidian University, Xi'an 710071, People's Republic of China

*Corresponding authors. E-mail: ningj@xidian.edu.cn (Jing Ning); chankwang@xidian.edu.cn (Dong Wang)

S1 Synthesis Process of Ni_3Si_2/Graphene and NiOOH/Graphene

S1.1 Ni_3Si_2/Graphene

First, the surface of the Ni foam was sonicated in a 0.3 mol L^{-1} $(NH4)_2S_2O_8$ solution for 5 min to remove contaminants and oxides. After washing with analytical grade acetone, ethanol, and deionized water for 5 min each, the Ni foam was dried with nitrogen. Second, the cleaned Ni foam/Si (111) stack was placed into the quartz tube furnace of the CVD system at a base vacuum of ~0.1 Pa. Ultrahigh-purity hydrogen was injected into the furnace tube at a flow rate of 10 sccm. Subsequently, the nickel foam was heated from 30 to 1030 °C at a heating rate of 20 °C min^{-1}. Once the temperature reached 1030 °C, ultrahigh-purity methane was introduced into the furnace tube (flow rate of 50 sccm) as the carbon source for 2 h. Third, the sample was cooled to 1000 ℃ and annealed for 1 h in a mixed atmosphere (10 sccm hydrogen and 800 sccm argon). The tube was then slowly cooled to ambient temperature at a cooling rate of ~5 ℃ min^{-1}. Finally, the obtained composite was washed three times with deionized water and ethanol, respectively and dried under vacuum at 80 ℃ overnight.

S1.2 NiOOH/Graphene

First, the surface of the Ni foam was sonicated in a 0.3 mol L^{-1} $(NH4)_2S_2O_8$ solution for 5 min to remove contaminants and oxides. After washing with analytical grade acetone, ethanol, and deionized water for 5 min each, the Ni foam was dried with nitrogen. Second, the cleaned Ni foam was placed into the quartz tube furnace of the CVD system at a base vacuum of ~0.1 Pa. Ultrahigh-purity hydrogen was injected into the furnace tube at a flow rate of 10 sccm. Subsequently, the nickel foam was

heated from 30 to 1030 °C at a heating rate of 20 °C min^{-1}. Once the temperature reached 1030 °C, ultrahigh-purity methane was introduced into the furnace tube (flow rate of 50 sccm) as the carbon source for 2 h. Third, the sample was cooled to 1000 ℃ and annealed for 1 h in a mixed atmosphere (10 sccm hydrogen and 800 sccm argon). The tube was then slowly cooled to ambient temperature at a cooling rate of ~5 ℃ min^{-1}. Forth, 100 mg urea was uniformly dispersed in 40 mL deionized water by stirring for 1 h. The mixture was then transferred into a 60-mL Teflon-lined stainless-steel autoclave and the fabricated graphene was added to the mixture. The autoclave was subsequently sealed and maintained at 180 °C for 2 h. Once the reaction was completed, the autoclave was cooled to room temperature. The final product was obtained after overnight drying under vacuum at 80 ℃.

We weighed the self-supporting Ni_3Si_2/graphene electrode to obtain a total electrode mass of 0.41 mg. Put the Ni_3Si_2/graphene electrode in 3 M HCl to have a full response. In the HCl solution, the reaction between Ni_3Si_2 and HCl is shown in the equation: $Ni_3Si_2 + HCl \rightarrow NiCl_2 + SiH_4\uparrow$ .Pick up the graphene floating on the solution, and washed it repeatedly with deionized water until the PH = 7. After drying for 24 h at 80 ℃ under vacuum, the mass of graphene weighed is 0.17 mg. Therefore, the mass of Ni_3Si_2 is 0.24 mg. After hydrothermal growth of NiOOH, the sample was clamped with tweezers and washed three times with deionized water and ethanol. The final product was obtained after overnight drying under vacuum at 80 °C. After weighing, the total mass of the Ni_3Si_2/NiOOH/graphene elecrode is 0.68 mg. Therefore, the mass of NiOOH is 0.27 mg.

Repeating this experiment process, we get that the total mass of the Ni_3Si_2/NiOOH/graphene electrode is about 0.7mg, the mass of graphene is about 0.18 mg, the mass of Ni_3Si_2 is about 0.26 mg, and the mass of NiOOH is about 0.26 mg.

S2 Supplementary Figures

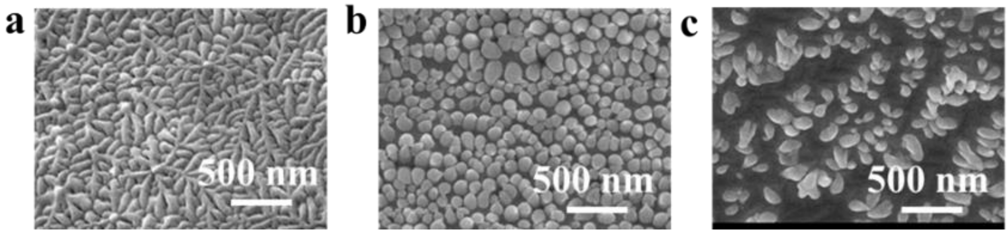

Fig. S1 SEM images of Ni_3Si_2 with different hydrogen flow rate. **a**) CH_4 : H_2 : Ar = 50 sccm: 10 sccm: 800 sccm, **b**) CH_4 : H_2 : Ar= 50 sccm: 25sccm: 800sccm, **c**) CH_4 : H_2 : Ar= 50 sccm: 50 sccm: 800 sccm

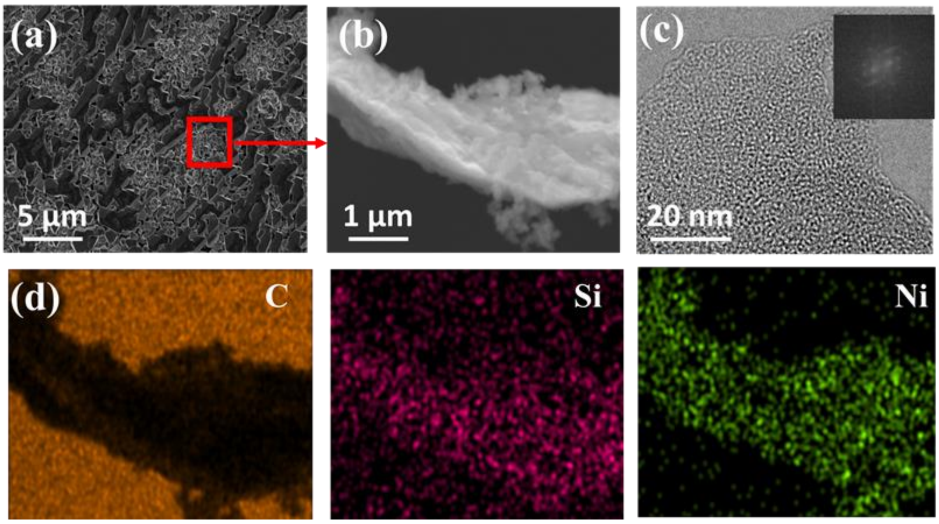

Fig. S2 a, b) SEM images of amorphous Ni_3Si_2. **c**) TEM image of amorphous Ni_3Si_2. **d**) EDS images of Ni_3Si_2 (C, Ni, Si elements)

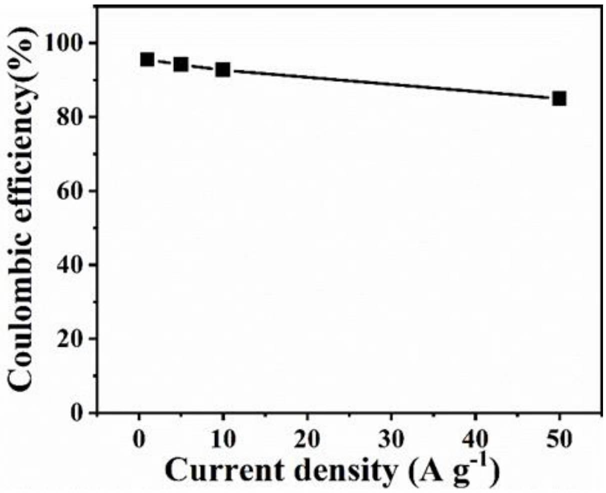

Fig. S3 Plot of Coulomb efficiency of Ni_3Si_2/NiOOH/graphene electrode

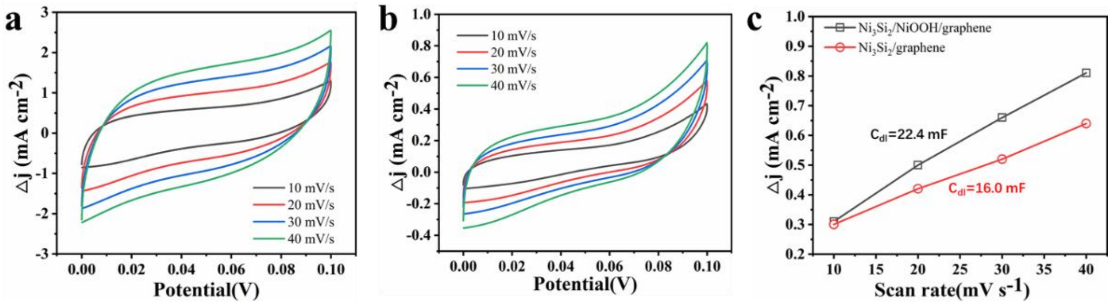

Fig. S4 a, b) CV plot of Ni_3Si_2/NiOOH/graphene and Ni_3Si_2/graphene. **c**) Double layer charging current vs. scan rate of Ni_3Si_2/NiOOH/graphene and Ni_3Si_2/graphene

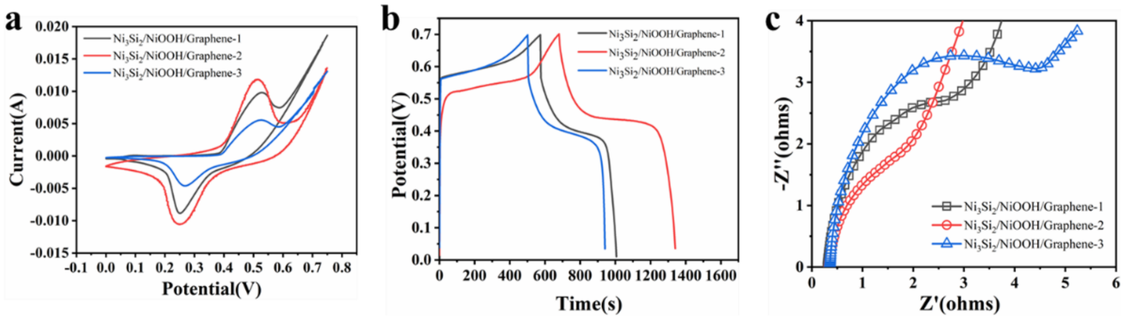

Fig. S5 a) CV curves at 10 mV s^{-1}, **b**) GCD curves at 1 A g^{-1}, and **c**) Nyquist plots of Ni_3Si_2/NiOOH/graphene-1, Ni_3Si_2/NiOOH/graphene-2, and Ni_3Si_2/NiOOH/graphene-3

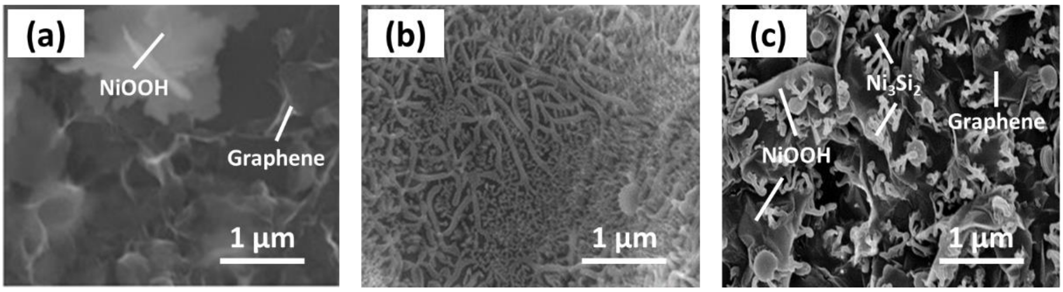

Fig. S6 SEM images of (**a**) NiOOH/Graphene, (**b**) Ni_3Si_2/Graphene, (**c**) Ni_3Si_2/NiOOH/Graphene

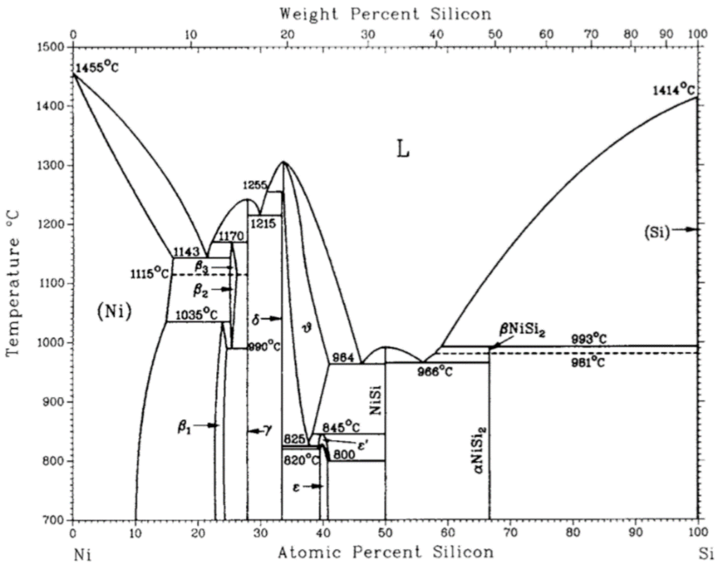

Fig. S7 Ni-Si binary diagram
